# Metrics to estimate differential co-expression networks

**DOI:** 10.1186/s13040-017-0152-6

**Published:** 2017-11-10

**Authors:** Elpidio-Emmanuel Gonzalez-Valbuena, Víctor Treviño

**Affiliations:** Cátedra de Bioinformática, Escuela de Medicina, Tecnológico de Monterrey, 64710 Monterrey, Nuevo León Mexico

**Keywords:** Differential correlation, Networks, Data simulation

## Abstract

**Background:**

Detecting the differences in gene expression data is important for understanding the underlying molecular mechanisms. Although the differentially expressed genes are a large component, differences in correlation are becoming an interesting approach to achieving deeper insights. However, diverse metrics have been used to detect differential correlation, making selection and use of a single metric difficult. In addition, available implementations are metric-specific, complicating their use in different contexts. Moreover, because the analyses in the literature have been performed on real data, there are uncertainties regarding the performance of metrics and procedures.

**Results:**

In this work, we compare four novel and two previously proposed metrics to detect differential correlations. We generated well-controlled datasets into which differences in correlations were carefully introduced by controlled multivariate normal correlation networks and addition of noise. The comparisons were performed on three datasets derived from real tumor data. Our results show that metrics differ in their detection performance and computational time. No single metric was the best in all datasets, but trends show that three metrics are highly correlated and are very good candidates for real data analysis. In contrast, other metrics proposed in the literature seem to show low performance and different detections. Overall, our results suggest that metrics that do not filter correlations perform better. We also show an additional analysis of TCGA breast cancer subtypes.

**Conclusions:**

We show a methodology to generate controlled datasets for the objective evaluation of differential correlation pipelines, and compare the performance of several metrics. We implemented in R a package called *DifCoNet* that can provide easy-to-use functions for differential correlation analyses.

## Background

Differential expression is one of the most important tools to study variations in the behavior of cells between tissues, species, and diseases [1–3]. This is not only highly intuitive but is also suitable for testing in the laboratory by turning specific genes on or off. However, a plethora of methods have shown that differential expression alone is unable to characterize some observed phenotypes [2, 4]. This may be caused by genetic, environmental, demographic, and technical factors [5]. In addition, differential expression ignores that genes operate in coordination with other genes in interconnected and regulated networks [6]. Therefore, some methods to detect alterations in gene expression networks have been proposed [7–16]. These methods can be classified based on the type of detection made: (i) differential expressed networks, (ii) differential gene-gene connections, and (iii) differential co-expressed genes. For differential expressed networks, the main idea is the detection of differential scores, which are calculated from sets of genes using several metrics such as principal components [7] and *t*-test-like scores [8]. The sets of genes can represent networks, pathways, or common properties such as ontologies. Nevertheless, these approaches focus on pre-defined networks instead of detecting specific genes, making it difficult to translate results to testable assays in the laboratory. For the second type of detection, which focuses on differential gene-gene connections, the core idea is to detect pairs of genes that seem ‘connected’ in one condition and ‘not connected’ in another condition, where the ‘connection’ is usually established by correlation thresholding [11]. As with the first approach, the study of specific gene-gene connections is difficult because assays turning on or off one of the genes would inevitably disrupt other connections, and thus it is difficult to study specific connections in the laboratory. For the last type of detection, differential co-expressed genes, the central concept is the detection of genes whose co-expressed genes differ between experimental conditions [15, 16]. Similar to previous methods, co-expression is commonly defined by correlations above a particular threshold. In this context, either all possible connections of a gene can be analyzed or only those connections that are highly correlated. This type of detection focuses on the gene rather than on a specific network or a connection, as is the case with the previous approaches. This has the advantage of the ability to detect altered genes, which can be easily tested in the laboratory independently, whether the network is known or not.

For the above practical reasons, in this paper, we will focus on differential co-expressed genes. To date, there are some implementations of this concept [15, 16]. In general, the core idea of detecting genes whose correlations are altered between conditions is maintained between methods. Nevertheless, the published methods mainly differ in the implementation of the metric used to detect the altered genes. For example, one method focuses only on differential connections for gene *i* estimating ∆C_i_ = |d_i0_-d_i1_|, where d_ix_ is the number of co-expressed genes above a correlation threshold in each condition [16]; another method considers all correlations using ∆C_i_ = sum(sqrt(|sign(A_i,j_)*A_i,j_^2^-sign(B_i,j_)*B_i,j_^2^|)^ß^), where A_i,j_ and B_i,j_ represent the correlation coefficients for gene *i* with all genes *j* under the A and B experimental conditions, sqrt is the square root function, ß weights for large correlation differences, and the sum function sums over all *j* genes [15]. Moreover, although other metrics have been proposed for differential networks instead of differential co-expression such as in CoXpress [8], one may ask whether those metrics are more effective. Indeed, we may propose other metrics to evaluate the difference of co-expressed genes. However, it is uncertain which metric could be the best to detect differential co-expression networks because they have been applied only to specific datasets. In addition, some published packages such as DiffCorr [17] and DiffCoEx [15] do not allow the evaluation of other metrics.

Therefore, in this paper, we aim to objectively compare the performance of different metrics under a well-controlled environment and to make available a framework to evaluate other possible metrics. We compared two metrics already used and published in scientific articles that evaluate differential co-expression, but we also propose four novel metrics and generalize a framework for future metrics. Previous applications of differential co-expression focus on normal and tumor data [16]. Instead, to demonstrate an additional application of our framework, we show a basic analysis on breast cancer subtypes from The Cancer Genome Atlas (TCGA). The implemented R package, *DifCoNet*, is available on CRAN.

## Methods

### Evaluated metrics

The overall goal is to determine the level of alteration of the co-expressed genes between two conditions. These alterations represent functional changes in the operational network. The level of co-expression is estimated using Spearman correlation. To quantify the overall level of alteration per gene, a variety of metrics have been used [15, 16, 18–21] . Besides these, we are exploring some others that could be potentially useful. In general, we consider two experimental conditions A and B where the gene expression level of *n* genes has been measured.

**Metric 1: Difference in the number of correlations.** Under the assumption that low correlation values are due to random chance, it has been proposed that

$$ M{1}_i=\mid \#{a}_i-\operatorname{}\#{b}_i\mid \operatorname{} $$

where *#a*_*i*_ and *#b*_*i*_ are the number of correlations of gene *i* higher than *th*_*1*_ in their corresponding A and B conditions [16, 21].

**Metric 2: Kolmogorov–Smirnov distance.** The Kolmogorov–Smirnov test is a common non-parametric statistical procedure to determine whether two probability distributions differ significantly [18]. It measures the greatest distance *D* between the empirical cumulative distributions. Because the vector of all correlations of gene *i* will generate a probability distribution,

$$ M{2}_i=\underset{1\le k\le n}{\max}\mid F{\left({a}_i\right)}_k-\operatorname{}F{\left({b}_i\right)}_k\mid \operatorname{} $$

where *a*_*i*_ and *b*_*i*_ are the correlation vectors of gene *i* in corresponding conditions, and *F*() is the empirical cumulative distribution function.

**Metric 3: Sum of large correlations differences.** M1 assumes that small correlations are random; instead, we can consider small differences in correlations as random, thus,

$$ M{3}_i=\sum \limits_{k=1}^n\left(|{a}_{i,k}-\operatorname{}{b}_{i,k}|\operatorname{}>{th}_3\right) $$

**Metric 4: Euclidean distance.** All the metrics above select specific correlations, which may potentially result in losing information; thus, a metric that uses all information such as the Euclidean distance could be powerful. We used a scaled version of the Euclidean distance to make the metric independent of the number of genes used:

$$ M{4}_i=\frac{1}{n}\sqrt{\sum \limits_{k=1}^n{\left({a}_{i,k}-{b}_{i,k}\right)}^2} $$

**Metric 5: Kullback–Leibler divergence.** In information theory, the Kullback–Leibler divergence is a measure of the difference between two probability distributions [19]. However, it is directional; thus, we used the sum of the two directions, similar to the Jensen–Shannon divergence [20], using

$$ M{5}_i=\sum \limits_{k=1}^nP\left({a}_{i,k}\right)\log \frac{P\left({a}_{i,k}\right)}{P\left({a}_{i,k}\right)}+\sum \limits_{k=1}^nP\left({b}_{i,k}\right)\log \frac{P\left({b}_{i,k}\right)}{P\left({b}_{i,k}\right)} $$

where *P*() is the probability function (zero probabilities are commonly ignored).

**Metric 6: Adjacency difference.** This metric was adapted from the *DiffCoEx* algorithm originally used to compute a matrix of adjacency differences [15]. It uses a β parameter to weight large correlation differences as.

$$ M{6}_i=\sum \limits_{k=1}^n{\left(\sqrt{\frac{1}{2}\left(|\operatorname{sign}\operatorname{}\left({a}_{i,k}\right){\left({a}_{i,k}\right)}^2-\operatorname{sign}\left({b}_{i,k}\right){\left({b}_{i,k}\right)}^2\operatorname{}|\right)}\right)}^{\beta } $$

Here, we used β = 2.5.

### Development of controlled data

**Fig. 1:**
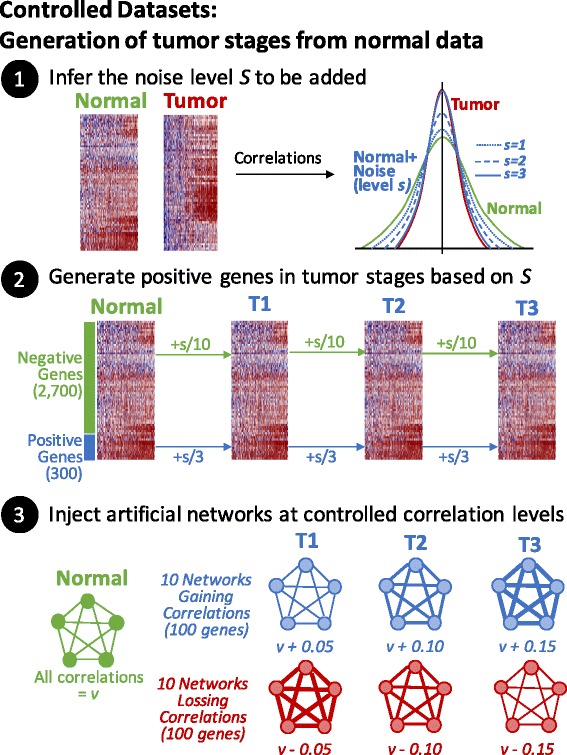
Generation of controlled datasets. The final dataset of 3200 genes contain 500 genes having altered correlations at different levels. From these, 300 of these genes gradually lose their correlations by adding noise (step 2). Also, 100 genes gradually increase their correlations (step 3 “Gaining Correlations”) while other 100 decrease their correlations (step 3 “Losing Correlations”). In this way, the dataset generated should not contain differential expressed genes

Experimental datasets may contain many differentially expressed genes, which introduces fluctuations that alter its correlations to other genes. In addition, the observed differential co-expression of experimental datasets is unknown and will inevitably be dependent on the metric used for detection. Therefore, a well-controlled simulated dataset is needed to evaluate the performance of the metrics. It is desired that simulated data maintains the complexity of the experimental data without being affected by intrinsic characteristics while still being capable of carrying the desired properties under study. Therefore, we will use gene expression data from normal tissues to generate artificial cancer progression stages by adding independent Gaussian noise at the gene level. In this way, the generated dataset will maintain the internal correlation structure but will not show differential expression [21]. Nevertheless, noise addition will reduce correlations, so we also used a second procedure to generate networks at the desired correlations levels. These procedures are described next and are summarized in Fig. 1. First, we selected 3000 randomly chosen genes from a gene expression dataset containing normal and tumor samples and standardized each subset (mean = 0 and standard deviation = 1 per gene). Second, we estimated the Gaussian noise level *s* that needs to be added to the normal data that resembles the correlation distribution of the tumor samples. Third, from the 3000 genes, we used 300 as *positive noised genes*, depending on the noise level *s*, to generate genes in tumor stages T1, T2, and T3 as follows: T1 = Normal + *N*(0, *s*/3), T2 = T1 + *N*(0, *s*/3), and T3 = T2 + *N*(0, *s*/3), where *N*(*m, sd*) is the normal function having mean *m* and standard deviation *sd*. Fourth, the remaining 2700 genes were used as *negative noised genes*, to which we added noise at a lower level to maintain variability as follows: T1 = Normal + *N*(0, *s*/10), T2 = T1 + *N*(0, *s*/10), and T3 = T2 + *N*(0, *s*/10). So far, the correlation structure of positive genes will resemble that of an observed tumor dataset, while the correlation structure of negative genes will be clearly less, similar helping to distinguish between both types of genes. Five, we added 200 additional genes arranged in 20 networks of 10 genes. Half of these networks were set to increase and the rest were set to decrease their correlation levels compared to normal data. For this, the R package *mvtnorm* was used [22]. This package generates multivariate random Gaussian datasets that follow a correlation structure from a given covariance matrix. Thus, for the networks, the covariance matrix *M* was defined as *M*_*i,j*_ = *v* for *i <> j* and *M*_*i,j*_ = 1 for *i* = *j*. For the normal dataset, the following values of *v* were used: {0.9, 0.75, 0.6, 0.45, 0.3}. Then, for the networks losing correlations in the artificial tumor stages, the correlations were set to T1 = *v −* 0.05, T2 = *v* − 0.1, and T3 = *v* − 0.15. Two networks of 10 genes were generated for each value of *v*. Similarly, to generate the networks that gain correlations, the *v* values used were {0.15, 0.30, 0.45, 0.60, 0.75} for the normal dataset, and the tumor stages were defined as T1 = *v* + 0.05, T2 = *v* + 0.1, and T3 = *v + 0.15*. We have used similar strategies to study physiological responses in biological networks [23].

### Estimation of statistical significance

A permutation-based approach is used to estimate the null distribution of the metric employed in the analysis [24, 25]. The *p*-value for a given gene is defined as the proportion of permutated metrics larger than the observed metric. When more than two conditions are analyzed, pairwise metric and *p*-value estimations are performed and the Fisher’s combined probability test is used to estimate an overall *p*-value. Finally, a false discovery rate approach is used to correct for multiple tests [26].

### Datasets

To compare the performance of the metrics, we used three cancer datasets from the Gene Expression Omnibus (GEO) at the National Center for Biotechnology Information (NCBI) containing at least normal and cancer samples and more than 60 samples in each group. The datasets chosen were GSE19804, GSE44076, and GSE25097 related to lung, colon, and liver cancer, respectively. The number of samples per group was 60, 96, and 243 (tumor and paired normal samples). For the metric comparisons, only normal data (standardized by gene) were used to generate controlled datasets. For this, the value of *s* was empirically estimated by testing a range of *s* values; the value whose correlation distribution most resembled the tumor correlation distribution was used. To show the application of our framework, we used the breast cancer dataset from TCGA to analyze the differences in correlations across molecular subtypes.

### Package implementation

**Fig. 2:**
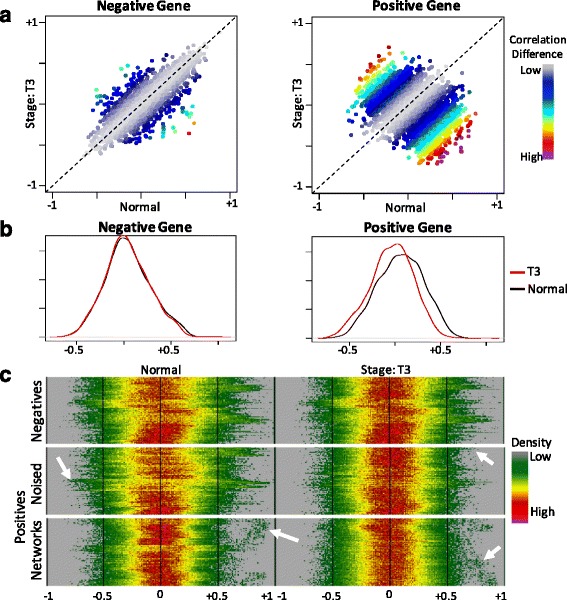
Validation of the controlled dataset. Panel **a** shows a comparison of all correlations of in normal versus tumor (stage T3) where differences in correlation are represented by colors codes as shown at right. Left and right show the differences in a negative gene and a positive gene respectively. Panel **b** shows the distribution of correlations of a representative negative and positive gene. Panel **c** shows a representation of distributions for negative and the two types of positive genes (by noise addition or network injection). Genes are shown in the vertical axis whereas the density of the distribution is represented by colors across correlation levels in the horizontal axis. For example, low density regions of the distribution, typically at both tails, are represented in dark green whereas high density regions are shown in yellow and red, typically close to 0. The arrows point to visible changes between normal and tumor T3

We implemented *DifCoNet* (DIFferential COexpression NETworks) in R, which is available in CRAN (https://cran.r-project.org/). Thus, the package can be easily installed using the *install.packages(“difconet”)* instruction in the R command line. The six metrics shown above are already implemented in *DifCoNet*. A user function receiving the correlation vectors of the two conditions can be specified to compute a distance metric not yet implemented. The main methods implemented in the *DifCoNet* package are related to (i) running the pipeline for estimating the differential co-expressed networks for a given dataset and corresponding parameters, (ii) displaying figures representing the differences in correlations (similar to those shown in Fig. 2), and (iii) generating a controlled dataset from a “normal” dataset (such as those used here). For this, the R functions *run.difconet, plot.gene.correlations*, and *build.controlled.dataset* are the corresponding functions implemented in *DifCoNet*.

## Results

### Validation of controlled datasets

Given that it is unknown which genes are differentially correlated in experimental datasets, it is necessary to begin with a dataset where negative and positive genes are well defined while still representing real biological scenarios. For this, we designed a computational procedure to generate controlled datasets containing specific changes in correlations based on a non-tumor dataset (Fig. 1). These datasets consist of a simulation of tumor progression starting with normal tissue data followed by a progression through three tumor stages (T1, T2, and T3). The last stage should contain the largest alterations in correlations whose overall correlation distribution is highly similar to the observed correlation distribution in the original tumor dataset. These datasets consisted of 3200 genes, including 200 genes that were generated by full-connected networks (considered positive) and 3000 genes that were randomly chosen from the normal tissue dataset, including 300 genes with higher levels of noise (also considered positive). All gene expression profiles were derived from a gene-standardized transformation of the normal data, and thus all genes should have mean and standard deviation equal to 0 and 1, respectively. By design, these controlled datasets should not contain differentially expressed genes. This was confirmed by a *t*-test, where no genes were called differentially expressed at FDR < 0.1.

To validate the generated datasets, we visually compared the correlations of putative positive and negative genes between the original normal dataset and the last tumor stage (T3). As shown in Fig. 2a, the difference in correlations is larger for a positive controlled gene than for a negative gene. These difference are also clear when comparing the distribution of correlations (Fig. 2b). Then, to generalize the visual comparison of several negative and positive genes at once, a heat map representation was used to summarize the correlation distributions (Fig. 2c). It is evident that positive genes have large alterations in the correlation distribution than do negative genes. Both types of putative positive genes are visible (generated by noise addition or network injection). These results show that controlled datasets seem to have clear alterations in the correlations of positive genes and scarce and random variations in negative genes. Therefore, these datasets can be used to compare the performance of different metrics for the detection of differentially correlated genes at different levels of alteration.

### Comparison of performance

To compare the six metrics while avoiding dataset-specific effects, we used three available lung, colon, and liver cancer datasets having a varied number of samples and gene expression platforms. In summary, the datasets contain normal genes, T1, T2, and T3 tumor stages, and 3200 genes of which 2700 should be negative genes and 500 should represent positive genes. The level of “positiveness” is low in T1, medium in T2, and high in T3. The details are described in the previous section and in the methods. We assessed all possible comparisons between normal and tumor stages. For the metrics that require a threshold (M1 and M3), we used 0.1, 0.3, and 0.5, adding the threshold value to the metric label. As a measure of performance, we counted the number of putative positive genes found in the top 500 genes ranked by the higher values of each metric.

The detailed results are shown in Tables 1, 2, and 3 for the lung, liver, and colon derived datasets.

**Table 1:** Observed positive genes from the Lung-based controlled dataset

Stages	M1.1	M1.3	M1.5	M2	M3.1	M3.3	M3.5	M4	M5	M6
N-T1	256	221	106	320	315	235	223	340	346	412
T1-T2	263	216	95	316	319	228	208	343	343	399
T2-T3	269	235	102	321	311	231	216	336	335	405
*Sensitivity at 33%*	*0.53*	*0.45*	*0.2*	*0.64*	*0.63*	*0.46*	*0.43*	*0.68*	*0.68*	*0.81*
N-T2	275	223	111	329	439	231	214	405	407	404
T1-T3	270	223	106	337	439	228	214	398	398	411
*Sensitivity at 66%*	*0.55*	*0.45*	*0.22*	*0.67*	*0.88*	*0.46*	*0.43*	*0.80*	*0.81*	*0.82*
N-T3	284	234	113	336	455	231	209	433	433	406
*Sensitivity at 100%*	*0.57*	*0.47*	*0.23*	*0.67*	*0.91*	*0.46*	*0.42*	*0.87*	*0.87*	*0.81*
Global Sensitivity	0.54	0.45	0.21	0.65	0.76	0.46	0.43	0.75	0.75	**0.81**
Relative Sensitivity	0.67	0.56	0.26	0.80	**0.94**	0.57	0.53	**0.93**	**0.93**	**1.00**

Sensitivity is shown in italics. Bold marks top values

**Table 2:** Observed positive genes from the Liver-based controlled dataset

Stages	M1.1	M1.3	M1.5	M2	M3.1	M3.3	M3.5	M4	M5	M6
N-T1	265	117	90	287	260	230	55	312	309	313
T1-T2	264	118	111	295	255	231	56	303	304	321
T2-T3	254	112	88	290	247	224	61	296	298	311
*Sensitivity at 33%*	*0.52*	*0.23*	*0.19*	*0.58*	*0.51*	*0.46*	*0.11*	*0.61*	*0.61*	*0.63*
N-T2	266	135	105	300	279	228	60	317	326	321
T1-T3	274	133	113	296	280	221	59	328	332	322
*Sensitivity at 66%*	*0.54*	*0.27*	*0.22*	*0.6*	*0.56*	*0.45*	*0.12*	*0.65*	*0.66*	*0.64*
N-T3	260	144	96	289	308	235	57	329	337	316
*Sensitivity at 100%*	*0.52*	*0.29*	*0.19*	*0.58*	*0.62*	*0.47*	*0.11*	*0.66*	*0.67*	*0.63*
Global Sensitivity	0.53	0.25	0.2	0.59	0.54	0.46	0.12	0.63	**0.64**	0.63
Relative Sensitivity	0.83	0.39	0.31	**0.92**	0.84	0.72	0.19	**0.98**	**1.00**	0.98

Sensitivity is shown in italics. Bold marks top values

**Table 3:** Observed positive genes from the Colon-based controlled dataset

Stages	M1.1	M1.3	M1.5	M2	M3.1	M3.3	M3.5	M4	M5	M6
N-T1	284	140	89	328	347	229	156	401	401	350
T1-T2	290	125	71	331	333	228	157	403	406	346
T2-T3	264	125	69	307	302	235	149	377	374	344
*Sensitivity at 33%*	*0.56*	*0.26*	*0.15*	*0.64*	*0.65*	*0.46*	*0.31*	*0.79*	*0.79*	*0.69*
N-T2	302	137	89	346	448	237	150	438	437	350
T1-T3	283	129	71	323	444	222	154	430	427	343
*Sensitivity at 66%*	*0.59*	*0.27*	*0.16*	*0.67*	*0.89*	*0.46*	*0.3*	*0.87*	*0.86*	*0.69*
N-T3	307	139	87	354	448	236	163	448	450	348
*Sensitivity at 100%*	*0.61*	*0.28*	*0.17*	*0.71*	*0.90*	*0.47*	*0.33*	*0.90*	*0.90*	*0.70*
Global Sensitivity	0.58	0.27	0.16	0.66	0.77	0.46	0.31	**0.83**	**0.83**	0.69
Relative Sensitivity	0.70	0.33	0.19	0.80	**0.93**	0.55	0.37	**1.00**	**1.00**	0.83

Sensitivity is shown in italics. Bold marks top values

**Fig. 3:**
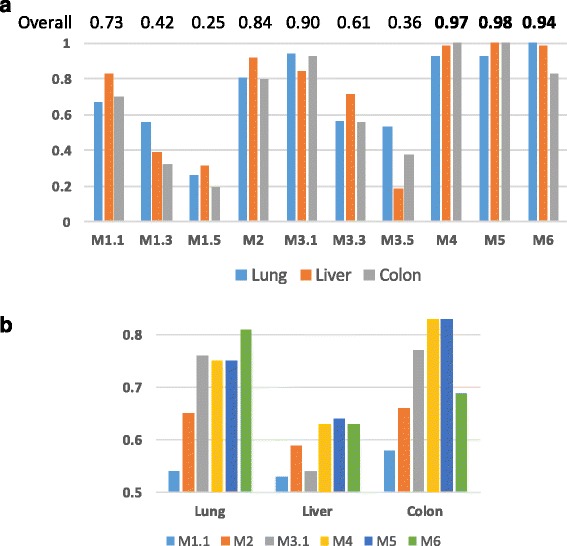
Comparison of the performance of metric across datasets. Panel **a** shows the relative sensitivity of all metrics in the three datasets. Panel **b** shows the concentrated global sensitivity per dataset. For M1 and M3, only the best threshold is shown

The maximum sensitivity reached was dependent on the dataset. We observed 0.81, 0.64, and 0.83 for lung, liver, and colon, respectively. For comparisons, we focus on the relative sensitivity, which was estimated by the percentage of detection relative to the maximum sensitivity observed in all metrics (Fig. 3). None of the metrics was the best in all datasets. The best overall performance was obtained by M5, but the differences between close competitors are very small (M4 and M6), and there were variations across datasets. We noted that the performance of M1 and M3 was the best when using lower thresholds, indicating that using more information (in lower thresholds) is better than less information (in higher thresholds). This supports the use of metrics that consider all available information. Overall, we observed that M4, M5, and M6 obtained similar and higher performances than M1, M2, and M3.

In this analysis, we also measured the sensitivity of all metrics during simulated tumor progression. That is, in our simulations, T1 carries only around 33% of the accumulated injected alterations, whereas T2 carries an accumulated 66%, and T3 reaches 100%. We noted that most of the metrics were also consistent across these increasing alterations. For example, in the lung dataset (Table 1), M4 reached 0.68 when detecting 33% of injected alterations (in T1), then increased to 0.80 for 66% of alterations (in T2), and then increased to 0.87 for 100% of the alterations (in T3). Similar behavior was observed across datasets and metrics, except for M6. For M6, the differences in performance between 33%, 66%, and 100% of the accumulated alterations were very small, indicating that M6 has more potential to detect subtle alterations than the other metrics.

We observed clear database effects. For instance, in the liver dataset, none of the metrics reached 70% global sensitivity, whereas, in the lung dataset, four metrics surpassed 70% and in the colon dataset, 3 surpassed 70% (Fig. 3b). In the colon dataset, two metrics surpassed 80% (M4 and M5).

### Comparison of estimations

**Fig. 4:**
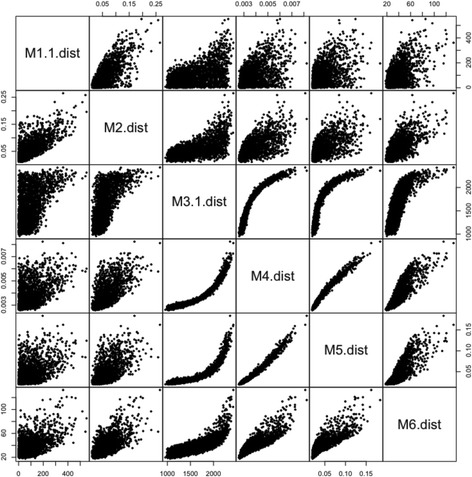
Comparison of metrics in the controlled dataset from Lung

The results described so far demonstrate that some metrics vary in their detection performance. These differences should correspond to different values of the metric, resulting in different prioritization of genes. To revise this, we compared the estimated values of the metrics between N and T3 in the controlled lung dataset. The results shown in Fig. 4 suggest that some clusters provide similar estimations. The clusters formed by metrics M3, M4, and M5 are clearer. M6 seems to be more similar to this cluster than to M2 and M1. The lack of similitude of M1 and M3 to the other metrics does not depend on the threshold used, as other thresholds provide similar results (data not shown). These results suggest that some metrics provide different priorities, thus suggesting that using metrics of different clusters could be convenient.

### Comparison of the running time

We estimated the time needed for each metric using a dataset containing 11,925 genes with two classes of 24 and 39 samples, respectively. The results shown in Table 4 clearly show that M4 = M3 < M1 < M6 < M5 < < M2. The running time of M1, M3, M4, and M6 is a few minutes, M5 takes more time, and M2 is close to hours. Thus, none of the metrics seem prohibitively slow, but this highlights differences of as much as one order of magnitude.

**Table 4:** CPU time needed per metric (in seconds)

Metric*	In Dataset	In 10 Permutations	Test Time
M1 (0.1)	16.2	204.5	220.7
M2	302.0	2975.1	3277.1
M3 (0.1)	8.8	158.6	167.4
M4	11.5	153.5	165.0
M5	63.5	679.8	743.3
M6	20.0	243.2	263.2

*The threshold used in shown between parenthesis

### Example of a differential correlation analysis

To show the potential and the implications of differential correlation analysis, we estimated the differential correlations across breast cancer subtypes from TCGA RNA-Seq data. In TCGA, the breast cancer subtypes are annotated according to molecular signatures of breast cancer [27] into Luminal A, Luminal B, Basal-Like, and Her2-Enrich (Normal-Like subtypes were removed due to the low number of samples). We made all pairwise estimations of differential correlations and further described selected comparisons. For this, from the 20,531 genes, we used 9981 that were in the top 25% of the highest mean or 25% of the highest standard deviation. We used metric 4 for these comparisons and the 484 samples with molecular classifications, distributed in 222, 118, 92, and 52 samples for Luminal A, Luminal B, Basal-Like, and Her2-Enriched subtypes, respectively.

**The differentially correlated but not differentially expressed genes can be enriched in specific functions.** We focused on those genes with exclusively differential correlation in any comparison but that will not be included in any differential expression analysis. Thus, we used those genes whose minimum *p*-value of differential correlation was lower than 10^−4^ and whose minimum *p*-value of differential expression was larger than 10^−4^. A DAVID analysis [28], which is focused on over-represented biological terms in a list of genes, revealed that the 694 differentially correlated analyzed genes are enriched in important biological functions (Table 5, overall). For example, fibronectins are known to play an important role in the interaction with stromal cells for extracellular matrix remodeling [29]. Our simple analysis revealed that, specifically, fibronectin type III is highly differentially correlated among those genes that are not differentially expressed. Overall, this analysis shows that differentially correlated genes may reveal interesting functions.

**Table 5:** Enrichment of biological terms in differential correlated genes between breast cancer subtypes

Analysis	Cluster Terms	Gene Counts Range	DAVID Enrich Score	*Benjamini *p*-Value
Overall	Signal peptide, Glycoprotein, Disulfide bond	155–199	6.18	1.3e-5
	Membrane	134–280	3.8	2.5e-3
	Fibronectin type III, FN3	17–21	3.41	8.7e-3
	Pleckstrin homology domain	22–30	3.41	2.4e-2
	Voltage-gated ch, Ion channel & Transport	15–33	2.96	6.3e-3
	Immunoglobulin I-set, subtype 2, domains	18–34	2.91	4.0e-3
	Innate immunity	20–25	1.96	1.1e-2
LumA-LumB	Mitosis, Cell Division, Cell Cycle	8–10	2.87	2.0e-3
	Secreted, Glycoprotein, Signal	21–30	2.56	7.4e-3
Basal-Her2	Palmitate, Lipoprotein, Receptor	6–8	1.76	1.1e-1
	NAD, Retinol metabolism	3–4	1.56	1.3e-1

*Minimum adjusted *p*-value reported in DAVID analysis

**Fig. 5:**
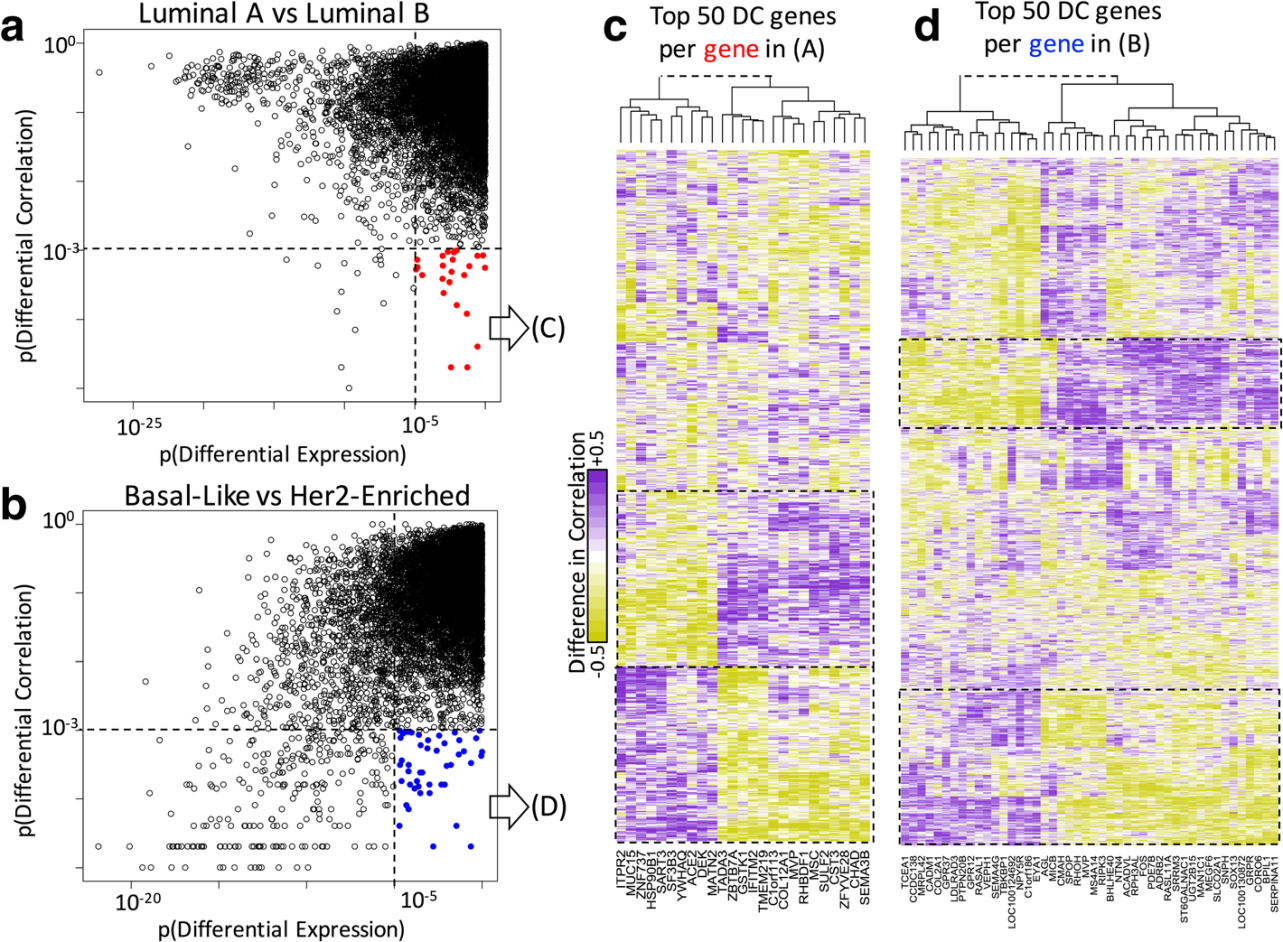
Analysis of specific subtypes in the TCGA breast cancer dataset. **a** Differential expression and correlation for the Luminal A and Luminal B comparison. The selected genes (in red) were further analyzed in panel (**c**). **b** Differential expression and correlation for the Basal-Like and Her2-Enriched comparison. The selected genes (in blue) were further analyzed in panel (**d**). **c** and (**d**) Genes whose difference in correlation is highest (in vertical) against the genes selected in (**a**) or (**b**) (in horizontal). Large network modules are framed by a dashed line. The difference of correlation is color-coded as shown in the left of (**c**) from −0.5 in greens to +0.5 in magentas. Genes in rows and columns were sorted according to hierarchical clustering using ward distance. The top trees were cut and joined by a dashed line for clarity

**The differentially correlated genes can gain or lose correlations with similar sets of genes.** We then made a similar analysis but focused on specific comparisons using the smallest and largest differences. These corresponded to the Luminal A compared with Luminal B subtypes and the Basal-Like compared with the Her2-Enriched subtypes, respectively. We wondered whether the genes that are losing or gaining correlation form tight network modules or broad gene-specific sub-networks. For this, we obtained the differentially correlated genes that were not differentially expressed (Fig. 5a-b); then, for each gene, we obtained the top 50 genes with the highest absolute difference in correlation. The results are presented in Fig. 5c and Fig. 5d, respectively. The figures show that, in both cases, the differentially correlated genes cluster together to form two large network modules. These modules are formed by a large fraction of all genes included in the comparisons, and they share a characteristic opposite trend where genes “disconnect” (losing correlation) from one module and “connect” (gaining correlation) in the other. Other network sub-modules can also be distinguished by specific groups of genes.

## Conclusion

The estimation of differentially correlated genes is an important feature for a deeper understanding of biological differences. Here, we studied several metrics to determine differential correlation under a highly controlled environment. We showed that there are differences in detection power and CPU time across diverse datasets. We also showed that some metrics are not correlated and could detect different sets of genes. Metrics that do not filter information seem to perform better. We showed a basic example demonstrating additional uses of differential correlation in a real dataset. Further, we implemented in R the *DifCoNet* package, which provides easy-to-use functions for differential correlation analyses.

## Abbreviations

GEO: Gene expression Omnibus
TCGA: The Cancer Genome Atlas
